# Effect of rotavirus vaccination on the burden of rotavirus disease and associated antibiotic use in India: A dynamic agent-based simulation analysis

**DOI:** 10.1016/j.vaccine.2024.126211

**Published:** 2024-09-17

**Authors:** Alec Gleason, Chirag K. Kumar, Eili Klein, Ramanan Laxminarayan, Arindam Nandi

**Affiliations:** a High Meadows Environmental Institute, Princeton University, Princeton, NJ, USA; b One Health Trust, Washington, DC, USA; c Department of Emergency Medicine, Johns Hopkins Bloomberg School of Public Health, Baltimore, MD, USA; d One Health Trust, Bengaluru, India; e Population Council, 1 Dag Hammarskjold Plaza, New York, NY 10017, United States

**Keywords:** India, Antimicrobial resistance, Rotavirus, AMR, Childhood diarrhea, UIP

## Abstract

**Background**: Rotavirus is a leading cause of diarrhea in infants and young children in many low- and middle-income countries. India launched a childhood immunization program for rotavirus in 2016, starting with four states and expanding it to cover all states by 2019. The objective of this study was to estimate the effects of the rotavirus vaccination program in India on disease burden and antibiotic misuse. **Methods:** We built a dynamic agent-based model of rotavirus progression in children under five within each district in India. Simulations were run for various scenarios of vaccination coverage in the context of India's Universal Immunization Programme. Population data were obtained from the National Family Household Surveys and used to calibrate the models. Disease parameters were obtained from published studies. We estimated past and projected future reduction of disease burden and antibiotic misuse due to full vaccination nationwide, by state, and by wealth quintile. **Results:** We estimate that rotavirus vaccination in India has reduced the prevalence of rotavirus cases by 33.7% (prediction interval: 30.7–36.0%), total antibiotic misuse due to rotavirus by 21.8% (18.6–25.1%), and total deaths due to rotavirus by 38.3% (31.3–44.4%) for children under five. We estimate total antibiotic misuse due to rotavirus infection to be 7.6% (7.5–7.9%) of total antibiotic consumption in this demographic versus 9.6% (9.4–9.9%) in the absence of vaccination. We project rotaviral prevalence to drop to below one case for every 100,000 individuals in those below five if vaccination coverage is increased by 50.3% (45.2–58.5%) to 68.1% (63.1–76.4) nationwide. **Conclusion:** Universal coverage of childhood rotavirus vaccination can substantially reduce inappropriate antibiotic use in India.

## 1 Introduction

Rotavirus is a leading cause of potentially fatal pediatric gastroenteritis, primarily in children under five years old [[1], [2], [3]]. Recent studies have also suggested a large, unrecognized disease burden in older children and adults [4]. There are potentially serious and lasting impacts of rotavirus on non-intestinal sites, as well [3]. Rotavirus is prevalent in most low- and middle-income countries (LMICs) [5], and is endemic in India [6], where the burden of disease is the second highest in the world after Nigeria [7]. Before India introduced a rotavirus vaccine in the national childhood immunization program (known as the Universal Immunization Programme or UIP) in 2016, conservative estimates indicated 11.37 million cases of rotavirus gastroenteritis and 78,000 associated deaths annually among under-five children [8]. However, most rotavirus cases lack gastroenteritis symptoms due to the immunity conferred from persistent reinfection [9,10].

India has made significant progress in providing routine childhood vaccinations to children under the age of five years. The launch of The Expanded Programme of Immunization (EPI) in 1978 followed by UIP in 1985 was instrumental in vaccination against tuberculosis, poliovirus, diphtheria, tetanus, and pertussis, leading to India achieving non-endemic status for poliomyelitis in 2012 [11]. In March 2016, a vaccine against rotavirus (ROTAVAC) was introduced in UIP for newborns in the states of Haryana, Himachal Pradesh, Andhra Pradesh, and Odisha, and its availability was gradually expanded to all states by 2019 [12,13]. ROTAVAC was based on India's indigenous strain 116E and created internally. It underwent a successful clinical trial in 2013 exhibiting outcomes similar to other commercially available rotavirus vaccines thus marking a significant accomplishment for the country [11,14]. While rotavirus vaccines have demonstrated high efficacy in high-income countries (HICs) [15,16], preliminary results from their use in LMICs including India have shown lower efficacy rates [17,18], in part due to complex and still not fully understood mechanisms of rotavirus transmission that complicate evaluation efforts [3]. Other proposed causes of reduced rotavirus vaccine efficacy include malnutrition, differences in breastfeeding patterns, exposure to intestinal pathogens, and co-administration with the Polio vaccine [19,20]. Rotavirus episodes have been additionally associated with antibiotic misuse in HICs [21], and even more so in LMICs [22]. Since antibiotics may be particularly harmful to young children and infants and their use can contribute to antimicrobial resistance (AMR) which is now a major global health challege [[23], [24], [25], [26], [27], [28], [29], [30], [31], [32], [33]], it is valuable to understand the extent to which antibiotic consumption can be reduced due to rotavirus vaccination among under-five children.

The objective of this study was to evaluate the effect of rotavirus vaccination program under UIP on disease burden and antibiotic misuse in under-5 children across India and quantify the potential benefits of increased vaccination in this age group over time. We performed a retroactive assessment of the vaccination rollout in India following its implementation and projected its impact for the future using state-wise, post-rollout data. We incorporated disease dynamics and thereby the benefits of herd immunity that largely influence disease transmission and burden [34]. In doing so, we overcome limitations of previously published models of rotavirus in India [[35], [36], [37], [38]]. The results of this study may set expectations for continued rotavirus vaccinations in India and implementations in other LMICs.

## 2 Methods

We developed an agent-based model (ABM) with agents representing the population of children under five years old in India, at the district level. ABMs are powerful tools in modeling infectious diseases as they permit interactions between individuals with heterogeneous characteristics (including movement patterns, behavior, and disease susceptibility) and their response to outside interventions. Therefore, it is possible to assess disease dynamics at higher resolution than through traditional approaches [39,40]. The characteristics of children were obtained from National Family Household Survey 2019–2021 (NFHS-5) data of 265,653 children under three years of age. These characteristics impact agent rotavirus vaccination status, probability to receive medical treatment when sick, and susceptibility to infection due to other factors. All additional parameters are derived from surveys and studies detailed in Table 1, aside from state-specific information. Given the vast number of states and districts, their characteristics are included in the model code repository.

**Table 1:** Model parameters.

Parameters	Value	Source
I. District attributes (values vary by district/state)		
Growth rate	-	NFHS-4 [43], NFHS-5 [44]
Population size	-	NFHS-4 [43], NFHS-5 [44] (approximated using growth rate and 2016 size)
Age distribution	-	NFHS-4 [43] (approximated using growth rate)
Wealth quintile distribution	-	NFHS-5 [44]
Proportion vaccinated	-	NFHS-5 [44]
Incidence of rotavirus	0.99 per child-year scaled state-wise by state disease positivity relative to average	Kumar et al. 2020 [45]

II. Basic individual characteristics		
Age	Sampled from associated district's age distribution	-
Wealth quintile	Sampled from associated district's wealth quintile distribution	-
Vaccination status	Probability based on proportion vaccinated in associated district	-
Vaccine efficacy (if vaccinated)	[0.5–0.64]	Jonestellar et al. 2017 [55]

III. Disease dynamics and infected characteristics		
Basic reproductive number		Gladstone et al. 2011 [9]. Best fit so that incidence of rotavirus across wealth quintiles 1 and 2 (two poorest wealth quintiles) matched incidence of rotavirus in Indian slums (0.99 cases per child-year) in the non-vaccination scenario
Incubation period (days)	[1–3]	“Rotavirus: Questions and Answers” [56]
Days infectious	Distribution same shape as Covid-19 infectious period distribution, centered at 12 days, the median rotavirus infectious period [The shape of covid infection was used as the shape for rotavirus infection is unavailable]	“Rotavirus” 2020 [57]
Seasonality effect on transmission	Scaled daily using averaged 2012–2016 disease positivity	Kumar et al. 2020 [45]

Infection mortality		
Probability of infected receiving clinic services	221/519 = 0.426	Gladstone et al. 2011 [9]
Mortality rate if receiving clinic services	58/8394 = 0.007	John et al. 2014 [8]
Days until death	Gamma distribution fitted to a median of 3 days and a range of 1–41 days	Sowmyanarayanan et al. 2012 [58]
Scaled mortality by wealth quintile		
Wealth quintile 1	2.33/mean(2.33,1.99,1.56,1.17,0.69) =1.505	Rheingans et al. 2012 [54]
Wealth quintile 2	1.99/mean(2.33,1.99,1.56,1.17,0.69) =1.286	
Wealth quintile 3	1.56/mean(2.33,1.99,1.56,1.17,0.69) =1.008	
Wealth quintile 4	1.17/mean(2.33,1.99,1.56,1.17,0.69) =0.756	
Wealth quintile 5	0.69/mean(2.33,1.99,1.56,1.17,0.69) =0.446	

Scaling on probability of receiving clinic services by age		
<2 years old	0.882/0.5 = 1.764	John et al. 2014 [8].
2+ years old	(1–0.882)/0.5 = 0.236	
Reinfection probability		
One past infection by the same serotype	[0.53,0.71]	Gladstone et al. 2011 [9]
Two past infections by the same serotype	[0.41,0.57]	
Three or more past infections by the same serotype	[0.26,0.41]	

Relative probability to be infected (inverse immune system strength) by wealth quintile		
Wealth quintile 1	1/(1–0.106 + 1–0.0795 + 1–0.053 + 1–0.0265 + 1) = 0.211	Church et al. 2019 [49], assuming that hygiene is directly related to wealth quintile; intermediate values between lowest hygiene and highest hygiene group are interpolated assuming there is a linear relationship between hygiene and immune system strength.
Wealth quintile 2	(1–0.0265)/(1–0.106 + 1–0.0795 + 1–0.053 + 1–0.0265 + 1) = 0.206	
Wealth quintile 3	(1–0.053)/(1–0.106 + 1–0.0795 + 1–0.053 + 1–0.0265 + 1) = 0.200	
Wealth quintile 4	(1–0.0795)/(1–0.106 + 1–0.0795 + 1–0.053 + 1–0.0265 + 1) = 0.194	
Wealth quintile 5	(1–0.106)/(1–0.106 + 1–0.0795 + 1–0.053 + 1–0.0265 + 1) = 0.189	

Rate of waning immunity		
Following one past infection	1/39 per week	Pitzer et al. 2011 [48]
Following two past infections	1/57 per week	
Diarrheal symptoms		
Probability of infected to have diarrhea	282/519	Gladstone et al. 2011 [9]
Scaled probability to have diarrhea by age		
<2 years old	0.882	John et al. 2014 [8]. The increased likelihood to have a severe infection requiring hospitalization is assumed to be the same as the increased likelihood to experience symptoms
2+ years old	0.118	

Antibiotic consumption		
Probability to consume antibiotics if diarrhea is present	0.189	Lewnard et al. 2020 [22]
Scaling for antibiotic consumption by wealth quintile		
Wealth quintile 1	0.462/mean(0.462,0.492,0.517,0.567,0.418) = 0.941	Allwell-Brown et al. 2021 [53]
Wealth quintile 2	0.492/mean(0.462,0.492,0.517,0.567,0.418) = 1.002	
Wealth quintile 3	0.517/mean(0.462,0.492,0.517,0.567,0.418) = 1.053	
Wealth quintile 4	0.567/mean(0.462,0.492,0.517,0.567,0.418) = 1.154	
Wealth quintile 5	0.418/mean(0.462,0.492,0.517,0.567,0.418) = 0.851	
Vaccine effectiveness against use of antibiotics		
0 to <2 years of age	[0.049–0.13]	Lewnard et al. 2020 [22]
2 to <5 years of age	[−0.073–0.13]	

IV. Travel characteristics		
Trip outside of district (followed by return)		
Probability to travel on any given day	0.005	OECD.Stat used for 2019 domestic travel estimate [47], World Bank Open Data used for 2019 population estimate [46]
Duration of trip (days)	[1–14]	Assumption

All agents in the same district can potentially interact with each other in a district-level network, with probabilities dependent on their age and wealth quintile. Children are born into the simulation and exit upon turning five years old. Children occasionally travel and enter other district networks, based on India's 2019 pre-Covid domestic travel rates (39,40). Children experience the following general states: uninfected, infected and asymptomatic, infected and symptomatic, or dead.

The model was run for multiple years with a time step of one day, until a steady state was reached. *The* model was initialized with a population size of 100,000 children and run across 100 simulations for three vaccination scenarios: 1) vaccination was never implemented, 2) vaccination was implemented based on NFHS 2019–2021 (NFHS-5) data, 3) vaccination coverage is steadily increased (until the disease becomes indetectable in the model). Running simulations with greater than a 100,000 population starting size is not feasible, as it exceeds 100GB of memory. However, at large population sizes, such as these, further increases have a negligible effect on model results.

The model was programmed in Julia using the Julia Agents package [41,42]. Model dynamics and parameters are detailed below.

### 2.1 General assignments by district

Children under five years old were assigned to districts proportional to each district's real population size as recorded in NFHS-4. Personal attributes, such as age and wealth quintile, were sampled from distributions of demographic data reflective of each district [43,44]. Vaccination status was also assigned based on recorded state-level vaccination coverage by wealth quintile according to the 2019–2021 NFHS (NFHS-5).

At the onset of each simulation, children were randomly infected to match the estimated prevalence of rotavirus in each state, which was calculated using incidence per year values, scaled by rotavirus positivity by state and the average duration of the pathogen's presence in hosts in our model (17.6 days) [9,45]. The number of infected individuals in each state at simulation onset was set to 17.6/365*state disease incidence. During model simulations, children were continuously added to districts based on each district's projected growth rate (birth rate minus death rate, estimated using NFHS-4 and NHFS-5 differences in population size). All simulations begin with an unrecorded one-year burn-in period to acclimate the population to the disease.

### 2.2 General transmission dynamics

The rotavirus transmission rate of the infected children varies with each day of their infectious period (i.e., increases until peak transmission, then gradually decreases). While those infected mostly spread disease within their district, both sick and healthy individuals will sporadically move to another district within their state or outside of their state and then return (with 0.5% chance on any given day, as approximated based on pre-Covid rates of domestic travel [46,47].

The sum of the transmission rates across all days of a sick child's infectious period is equal to the child's assigned reproductive number. The reproductive number for each infected agent is scaled by a) the total number of contacts the infected individuals have in the district. Furthermore, the transmission rate of all infected children is scaled by day of the year based on the seasonality of the virus observed in India [45].

The probability for a healthy individual to become infected after exposure to someone sick is attenuated by immunization, which occurs through vaccination and/or recovery from past infection.

### 2.3 Reinfection

If an individual who was previously infected comes in contact with the virus, the individual has some level of immunity, decreasing the probability of becoming infected. We assign reinfection immunity following one reinfection, two reinfections, and three reinfections as measured in a cohort in India [9], Reinfection immunity wanes with time according to a rate estimated by another modeling study [48]. In our model, we also account for the difference in rates of infection between exposed individuals across wealth quintile due to hygiene differences that affect immune system strength [49].

### 2.4 Contact matrices

We used a study of high-resolution human mixing patterns to synthesize contact matrices for each district [50]. Since rotavirus transmission can occur through interpersonal contact from touch [51], and is hypothesized to spread through air [3,52], we incorporated frequency of contact as a determinant of total transmission. Contact matrices were created for each district based on a) population sizes of five age groups in the district (younger than one, between one and two, between two and three, and between four and five years old) relative to the total population of the district, b) district population size relative to the total population across all districts, and c) the land area of the district relative to average district land area. In the contact matrix of district *k*, denoted as $F^{k}$, the recorded values that represent contact from an individual of age *i* with an individual of age *j* are:

(1.1) $$F_{\mathrm{ij}}^{k}=\frac{\frac{\phi_{i}^{k}\left(\phi_{j}^{k}-\delta_{\mathrm{ij}}\right)}{\nu^{k}-1}}{N_{i}}\cdot \frac{\alpha_{k}}{A}$$

where $F_{\mathrm{ij}}^{k}$ represents the per capita probability of contact from an individual of age *i* with an individual of age *j* in district *k*; $\varphi_{i}^{k}$ is the total number of people of age *i* in the district; $\delta_{\mathrm{ij}}$ is the Kronecker delta function (equal to one if *i = j*, zero otherwise); $\nu^{k}$ is the total number of individuals of all age groups in the district; $N_{i}$ is the total number of individuals of age *i* across all districts; $\alpha_{k}$is the area of the district, and A is the average area across all districts.

### 2.5 Assignment of reproductive number

The reproductive numbers of infected individuals are a) proportional to the rate of contact the individuals have with others within the same district, and, b) distributed with an average equal to the reproductive number of rotavirus. $F_{i}^{k}$ designates the relative probability of contact between an individual of age *i* and any other person in the same district, *k*, and is computed as:

(1.2) $$F_{i}^{k}=\sum_{j=0}^{12}F_{\mathrm{ij}}^{k}\cdot \frac{\varphi_{j}^{k}}{\nu^{k}}$$

The variables are the same as those defined in eq. 1.1. Using the relative probability of contact, $F_{i}^{k}$, the reproductive number for an infected individual of age *i* in district *k.*

is calculated as shown below:

(1.3) $$\frac{F_{i}^{k}}{\bar{F}}\cdot R_{0}$$

where value $F_{i}^{k}$ denotes the relative probability of contact with others for a child of age *i* in state *k*, $\bar{F}$ is the average probability of contact by state, and $R_{0}$ is the basic reproductive number of rotavirus. The reproductive number was tuned so that the incidence of rotavirus in the model scenario with no vaccination matched recorded values in India [9].

### 2.6 Antibiotic use

We scaled the probability for a child under five years old with rotavirus to experience symptoms or be asymptomatic (Table 1) by the status of whether the child is below two years old, as such children are 88% more likely to have a severe infection requiring hospitalization [8,9]. In the case that an infected child experiences diarrheal symptoms, we used estimates of antibiotic-treated rotaviral diarrhea in young children [22], scaled by a) the relative difference in consumption of antibiotics by wealth quintile in Southeast Asian LMICs [53], and, b) the reduced likelihood of taking antibiotics if vaccinated against rotavirus [22]. We designated the resulting value as the probability for the child to receive antibiotics. We account for the effect of vaccination on reducing antibiotic use by a) preventing infection and thus antibiotic use, and b) preventing symptoms in infected children and thus antibiotic use.

### 2.7 Mortality

We used the probability for an infected child under five years old to be treated at healthcare facilities using a surveillance study to determine whether a child receives medical care [9]. In the case that a child received treatment, we assigned the likelihood for the child to die based on the mortality rate following a healthcare visit, scaled by the relative difference in mortality due to rotavirus between wealth quintiles (in the case that vaccination status does not differ by wealth) and the status of whether the child is under two [8,54], as 88% of those hospitalized with rotavirus are under two years of age [8]. The scaling by wealth quintile captures the effect of differences in access to resources and sanitation, among other factors that vary by wealth, on a child's chance to experience mortality.

### 2.8 Model scenarios

Using rotavirus incidence data across 2011–2013 which we scaled by state via state disease positivity data [8,45], we retroactively modeled rotavirus progression and disease burden from 2012 to 2022 under two scenarios: a) with vaccination implemented as it had been starting in 2016, and b) without vaccination having ever been implemented (as a baseline for comparison). Using outputs of disease prevalence from the retroactive model with vaccination, we simulated a third scenario: the proactive progression and disease burden of rotavirus (through 2034) with vaccination coverage gradually increasing by day. We modeled ten years of increasing vaccination coverage, followed by a two-year period of full coverage.

All model parameters are outlined in Table 1, aside from state- and district-specific parameters available in the model code repository.

### 2.9 Estimating the proportion of total antibiotic use attributable to rotavirus

The median number of defined daily doses (DDD) of antibiotics used by 1000 people per day was approximately 10.7 across the years 2011 to 2019 in India [59]. Since population antibiotic consumption was computed based on DDDs, a metric used for adults, no differentiation was available between child and adult antibiotic consumption [59]. Using Global Burden of Diseases 2019 (Institute of Health Metrics and Evaluation) data for the incidence of febrile, respiratory, and diarrheal diseases frequently treated with antibiotics in India across all age groups, and ages less than five years old [7], we found incidence of such diseases in the below-five category to be about 1.15 more frequent than in the population as a whole. Thus, we estimate there to be 1.15 * 10.7 = 12.3 DDD per 1000 children under five years old per day, assuming a child under five is as likely to take antibiotics during infection as the general population (due to limited available data in the literature). We multiplied median model estimates of antibiotic courses provided per child per year by the value of five to simulate five treatment days per antibiotic course (5 DDD) and divided the resulting value by 12.3.

### 2.10 Estimating herd effects on immunity with rotavirus vaccination

To estimate herd effects of immunity following vaccination rollout, we subtracted the expected direct effect of the rotavirus vaccine from the observed reduction in model infection incidence between vaccination and non-vaccination scenarios at steady state. The direct effect of rotavirus vaccination was calculated as the product of vaccination coverage and vaccine efficacy.

### 2.11 Statistic reporting

All model simulations are run one hundred times, and median statistics are reported. 95% prediction intervals are generated using 2.5th and 97.5th percentile results.

## 3 Results

### 3.1 Current vaccination coverage

**Fig. 1:**
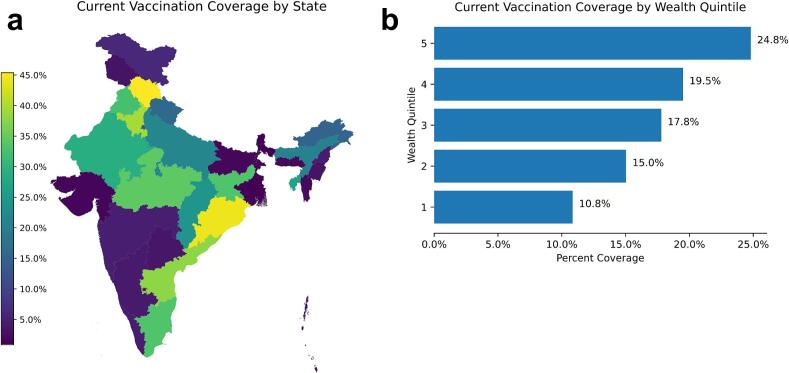
Current vaccination coverage by state and wealth quintile.

According to NFHS-5, current full (three-dose) rotavirus vaccination coverage of children under five years old is highly variable, both by state and by wealth quintile. A band across India comprising Northern states and low-Eastern states has the highest rates of vaccination, ranging from 21.5% in Uttar Pradesh to 45.4% in Himachal Pradesh. The outlying states have the lowest coverage – from as low as 0.8% in West Bengal (Fig. 1A). Coverage by wealth quintile increases in a gradient fashion, rising from about 10.9% in quintile one to 24.8% in quintile five (Fig. 1B).

### 3.2 Estimates of averted disease burden and antibiotic misuse due to current vaccination coverage

Estimates of reduced disease prevalence, incidence, antibiotic misuse, and deaths are stratified by state and wealth quintile and are available in supplementary materials (tables S1 and S2).

**Fig. 2:**
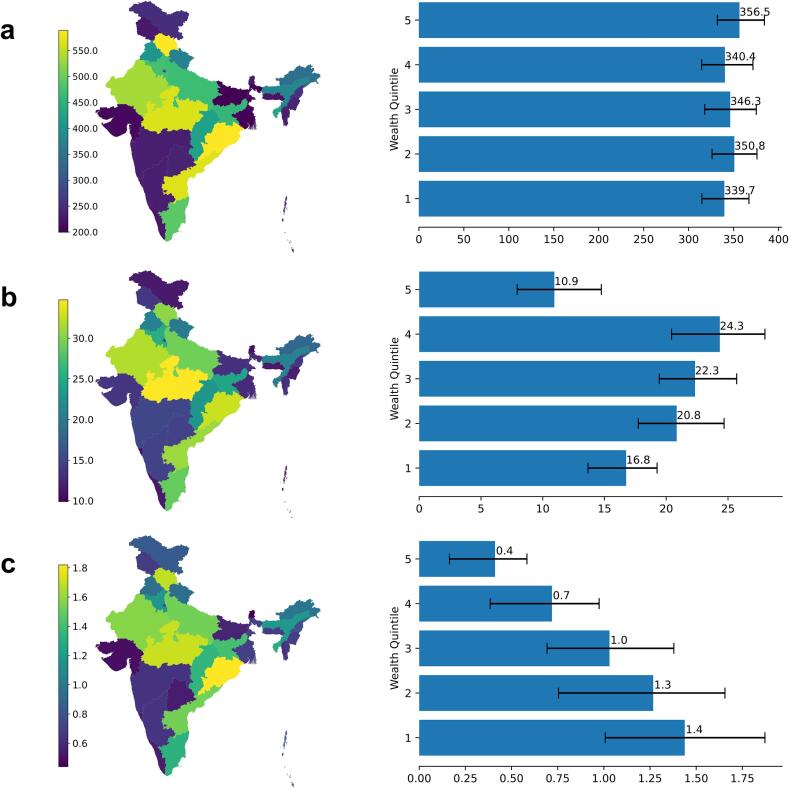
Reduced rotaviral disease burden and antibiotic misuse with current vaccination coverage. Results are reported per 1000 children per year by state and wealth quintile for A) reduced cases B) reduced misused antibiotic courses, and, C) reduced deaths. Error bars indicate 95% prediction intervals.

According to our estimates, current (2023) levels of vaccination coverage have significantly reduced the burden of rotavirus by state and wealth quintile. The band of states with the highest vaccination coverage experienced the most benefit. The reduction in rotavirus cases per 1000 children per year ranged from a minimum of 199.5 (95.5–285.3) in Bihar to a maximum of 588.6 (497.6–659.5) in Himachal Pradesh (Fig. 2A). The reduction of antibiotic misuse per 1000 children per year ranged from 9.9 (−2.1–24.7) antibiotic courses in Lakshadweep to 34.7 (24.9–45.0) in Madhya Pradesh. The reduction of deaths per 1000 children per year ranged from 0.4 (−0.5–1.8) deaths per 1000 children in Sikkim to 1.8 (0.8–2.7) deaths per 1000 children in Madhya Pradesh.

**Fig. 3:**
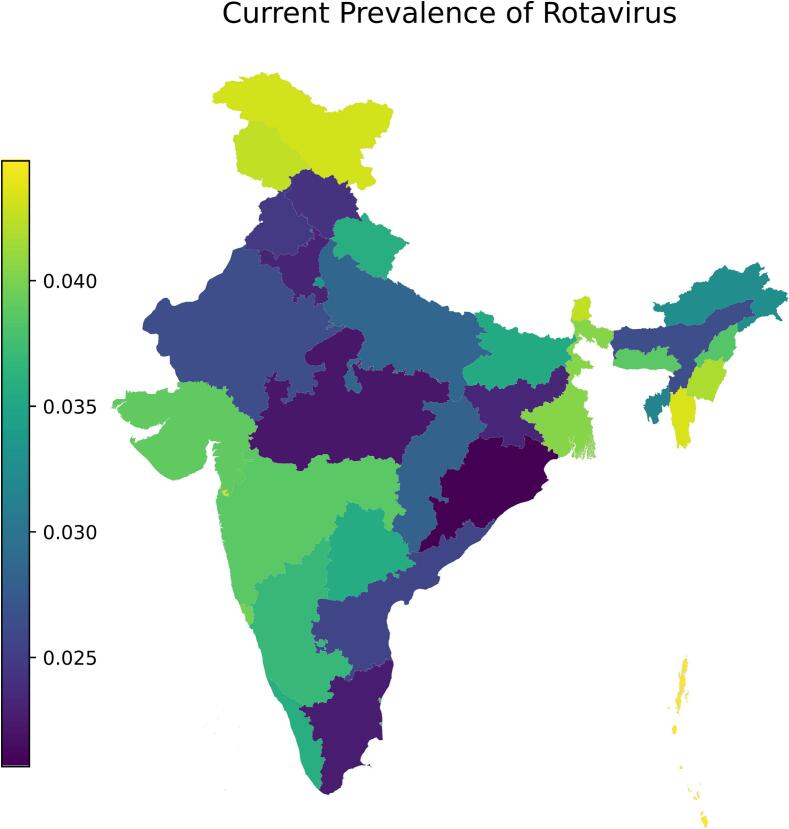
Estimated current prevalence of rotavirus. Results are reported as the proportion of infected children under five years of age (i.e., 0.020 corresponds to 2 out of 100 children infected) when rate of transmission is at its average rate for the year.

Compared to the highly heterogeneous distribution of reduced rotaviral cases per state, the reduced cases per wealth quintile are much closer. There is no statistical difference in the incidence of rotavirus between wealth quintiles 1–5 (Fig. 3A). However, reduced incidence of rotavirus in wealth quintile 5 is higher than that of all other wealth quintiles and has 2.9 (20.8–10.5) more reduced cases per 1000 children per year than quintile 1. In contrast, differences in reduced antibiotic misuse significantly vary across wealth quintiles (Fig. 3B, Fig. 3C). Wealth quintile 5 experiences the least reduction in antibiotic use at 10.9 (7.1–13.9) antibiotic courses per 1000 children per year, whereas quintile 4 experiences the greatest reduction of 24.3 (20.7–28.2). The difference in mortality due to rotavirus is significant between wealth quintiles as well (Fig. 3C). Wealth quintile 5 has the least reduction of 0.4 (0.42–0.7) deaths per 1000 children per year versus wealth quintile 1 with the greatest reduction of 1.4 (1.0–1.9).

Overall, we estimate current (2023) levels of vaccination coverage have reduced rotavirus prevalence (the proportion of children positive for rotavirus) nationwide by 33.7% (30.7–36.0%). Included in this value, herd effects of immunity account for 23.6% (19.5% - 26.9%) protection, while the direct effect of vaccination (i.e. benefits to the vaccinated individuals) represents the rest. These results are consistent with herd effects of immunity estimates in other LMICs [60]. We include a map with estimations of the current rotavirus distribution by state (Fig. 3). Additionally, we project total antibiotic misuse due to rotavirus to have been reduced by 21.8% (18.6–25.1%), and total deaths due to rotavirus by 38.3% (31.3–44.4%) for children under five years old. Using median drug consumption values from 2011 to 2019 [61], scaled to reflect increased consumption in children under five (see methods), we estimate total antibiotic usage due to rotavirus infection in this age group to be 7.6% (7.5–7.9%) of total antibiotic consumption – a number that otherwise would have been 9.6% (9.4–9.9%) if vaccination had not been implemented.

### 3.3 Projected averted disease burden and antibiotic misuse with increased vaccination

We found that uniformly increasing vaccination coverage across all states by 50.3% (45.2–58.5%) to 68.1% (63.1–76.4%) resulted in the reduction of rotavirus cases to undetectable levels in our model population of 100,000 children under five. This means that the national prevalence of rotavirus originating from this demographic was reduced to below one in every 100,000 individuals. We estimate herd effects of immunity account for about 60.8% (50.3–68.5%) of the total 100% reduction in detectable cases.

## 4 Discussion

Prior to vaccination, rotavirus incidence in India averaged approximately one case per child per year [9]. Our estimated national drop of 0.34 cases per child per year is a significant decrease, considering only about 17.8% current national vaccination coverage against rotavirus according to the 2019–2021 NFHS. There is also great variation in reduced disease incidence across states, corresponding to vaccination coverage (Fig. 1A, Fig. 2A). According to our estimates, states that had very low coverage still moderately benefitted from vaccination in neighboring states due to reduced interstate transmission of the virus. While there are not many available studies on the results of current rotavirus vaccination in India, our findings are consistent with surveillance study estimates that rotavirus vaccination has decreased rotaviral diarrhea incidence by 15.6% to 78.6% [62].

There is no significant difference in reduced disease incidence between most wealth quintiles (Fig. 2), which is consistent with other studies noting similar spread of rotavirus between wealth quintile [63,64], thus terming it a ‘democratic virus.’ [63] Although lack of hygiene and overcrowding have impact are reflected in our model via state-specific rotavirus incidence (using state disease positivity data) [45], it is minimal [49]. This unique feature sets rotavirus apart from other diarrheal diseases as rotavirus may not just spread through hand-to-mouth contact, but may be airborne as well [65,66]. Indeed, rotavirus seasonality trends are similar to that of respiratory diseases [66]. Despite higher vaccination coverage in wealthier quintiles, the effects of herd immunity reduce the likelihood of infection in different wealth groups by a similar margin in our simulations.

Irrespective of the relatively small difference in disease spread between all wealth quintiles, there is large disparity in disease burden and antibiotic misuse across these groups due to different trends in antibiotic use and access to resources. According to a systematic analysis of Southeast Asia surveys largely driven by India trends [59], wealth quintile 5 consumed the least antibiotics in 2017 (close in value to quintile 1), whereas quintile 4 consumed the most. These trends are reflected in our model estimates of reduced antibiotic misuse by wealth quintile (Fig. 2B). Differential access to healthcare resources and hygiene also largely influences the difference in averted mortality by wealth quintile despite similar rotavirus incidence. As poorer quintiles experience higher mortality rates than wealthier quintiles upon infection, the same degree of protection against disease across wealth quintiles from vaccination (same number of averted cases per population unit) should result in more deaths averted per population unit in poorer quintiles. Since states are comprised of varying proportions of each wealth quintile, we see slight differences in patterns of reduced incidence, antibiotic misuse, and mortality estimates between states (Fig. 2) that are due to wealth quintile differences in access to health resources, hygiene, and antibiotic use.

An important and possibly underappreciated effect of rotavirus vaccination is the extent of averted antibiotic misuse. Given particularly harmful effects of antibiotics on young children [[24], [25], [26], [27], [28], [29], [30], [31], [32], [33]], the estimated reduction in rotavirus-driven antibiotic use in India from 9.6% to 7.7% of total antibiotic consumption in this demographic (a drop of total antibiotic use by 1.9%) is particularly significant. According to our predictions, if vaccination coverage levels are uniformly increased to 68.2%, antibiotic consumption in this demographic would be reduced by close to 9.6%, representing additional and potentially vast averted disease burden from antibiotic misuse.

In 2019, an estimated 1.3 million deaths worldwide were attributable to AMR [67]. By 2050, AMR is projected to cause 10 million deaths per year globally [68], with a cumulative associated economic cost of $100–$210 trillion [69]. Antibiotic overuse, including misuse for viral diseases such as rotavirus infection and certain respiratory illnesses, remains a major driver [[70], [71], [72], [73]]. Our findings show that large-scale national vaccination efforts can provide substantial global health benefits by reducing antibiotic overuse and associated progression of AMR [22].

To the best of our knowledge, previously published modeling of rotavirus in India is limited to five studies [[35], [36], [37], [38],^74^]. However, four do not simulate disease dynamics, thus not accounting for various factors affecting disease burden [35,36,38,74]. This includes highly impactful herd immunity. While one does account for disease dynamics [38], it does not incorporate state-level properties, wealth quintile characteristics, or seasonality. No paper evaluates the reduction of antibiotic misuse due to rotavirus vaccination. Also, importantly, three studies were published two years prior to India implementing rotavirus vaccination [[35], [36], [37]], and one was published in the second year of the four years that it took for vaccines to be distributed across all states [38]. Therefore, reliable post-vaccination data was not available for these studies. The most recent study [74], which was published after the vaccine rollout, only reports reduced odds of diarrhea following vaccination. No standard burden of disease estimates are provided, such as reduced disease incidence and mortality). In this paper, we included the most recent available values for herd immunity, state- and wealth quintile-level variables, seasonality, and additional variables not found in past modeling (Table 1). Due to the differences in time periods, contributing factors, and output characteristics between our model and previously published studies, model results were not compared between studies.

There are some limitations to our analysis. We only consider rotaviral transmission and disease burden originating from children under five. While this is a particularly affected age group, older individuals get rotavirus at lower rates [75]. Disease that spreads to younger individuals from older individuals is unaccounted for. Furthermore, we assume equal efficacy of vaccination against each rotavirus variant. We do not account for the evolution of the virus over time, with potential corresponding change in vaccine efficacy. Additionally, there are still poorly understood features of rotavirus, such as the level and duration of cross-immunity derived from various strains and changes in immune response over time following vaccination [3,76]. Due to limited available data, we also assume that children under five are as likely to consume antibiotics during disease as is the general population. These limitations may render our estimates conservative.

Despite these limitations, our results highlight the significant effect of rotavirus vaccination on reducing disease burden and antibiotic misuse in India. It is necessary to continue expanding rotavirus vaccination coverage and evaluate various strategies for improving the efficiency of UIP. Recent health technology advancements in India such as the electronic vaccine intelligence network (eVIN) and vaccination delivery platforms (Co-WIN and U-WIN) will be particularly instrumental in streamlining these efforts [77]. According to a recent review of the rotavirus vaccination program in India [78], evaluation studies such as ours motivate healthcare providers and parents to support the ongoing vaccination efforts. Our data may further assist other LMICs lacking sufficient resources to assess potential reduced disease burden via rotavirus vaccination [79].

## Code availability

The underlying code, data, and models for this study are available in GitHub and can be accessed via this link http://github.com/Agleason1/Agent-Based-Infection-Model--Rotavirus.

## CRediT authorship contribution statement

**Alec Gleason:** Writing – review & editing, Writing – original draft, Investigation, Formal analysis, Data curation. **Chirag K. Kumar:** Writing – review & editing, Writing – original draft, Methodology, Formal analysis, Data curation. **Eili Klein:** Writing – review & editing, Supervision, Methodology. **Ramanan Laxminarayan:** Writing – review & editing, Supervision, Investigation, Funding acquisition, Conceptualization. **Arindam Nandi:** Writing – review & editing, Supervision, Investigation, Funding acquisition, Conceptualization.

## Declaration of competing interest

All authors declare no financial or non-financial competing interests.

## Data Availability

Link for data and code repository has been included in tha manucript.

## Appendix A Supplementary data

Supplementary material 1 Reduced prevalence, incidence, antibiotic misuse, and deaths from current vaccination coverage. Image 1

Supplementary material 2 Rotavirus ABM graphs depicting median results from simulation of the years 2010-2022. Image 2
