# Supplementary material for: Effect of rotavirus vaccination on the burden of rotavirus disease and associated antibiotic use in India: A dynamic agent-based simulation analysis
Source: Vaccine. 2024 Sep 17;42(22):None. doi: 10.1016/j.vaccine.2024.126211 (PMC11385704; doi:10.1016/j.vaccine.2024.126211)
Supplement: Supplementary material 1 — Reduced prevalence, incidence, antibiotic misuse, and deaths from current vaccination coverage. [file mmc1.docx]

**Table S1. Reduced prevalence, incidence, antibiotic misuse, and deaths from current vaccination coverage by state.**

| State/Territory | Reduced Prevalence (% Reduction) | Reduced Cases per 1000 Children per Year | Reduced Antibiotic Courses per 1000 Children per Year | Reduced Deaths per 1000 Children per Year |
| --- | --- | --- | --- | --- |
| Andaman & Nicobar Islands | 19.5 (9.8, 34.1) | 227.4 (114.9, 396.5) | 10.7 (-2.1, 25.9) | 0.7 (-0.4, 1.6) |
| Andhra Pradesh | 51.6 (42.2, 60.6) | 567.9 (465.8, 666.8) | 31.0 (20.6, 42.0) | 1.5 (0.4, 2.4) |
| Arunachal Pradesh | 33.2 (23.6, 46.0) | 340.3 (242.9, 471.1) | 18.1 (7.1, 32.0) | 1.0 (-0.1, 2.3) |
| Assam | 40.5 (31.4, 51.5) | 379.9 (293.7, 482.2) | 20.9 (10.8, 32.7) | 1.2 (0.4, 2.0) |
| Bihar | 21.3 (10.1, 30.5) | 199.5 (95.5, 285.3) | 13.3 (3.0, 22.4) | 0.6 (-0.1, 1.3) |
| Chandigarh | 36.9 (25.5, 46.7) | 370.7 (256.9, 469.2) | 13.2 (1.3, 23.7) | 0.8 (0.0, 1.5) |
| Chhattisgarh | 41.9 (31.4, 51.9) | 424.4 (318.0, 525.5) | 23.0 (11.5, 33.5) | 1.3 (0.6, 2.2) |
| Dadra & Nagar Haveli and Daman & Diu | 19.3 (8.6, 31.8) | 218.0 (98.0, 358.0) | 10.8 (-0.6, 25.2) | 0.6 (-0.6, 1.7) |
| Delhi | 29.4 (15.9, 38.2) | 287.8 (156.6, 374.3) | 14.3 (-1.4, 23.4) | 0.7 (-0.1, 1.5) |
| Goa | 21.3 (11.8, 31.2) | 227.8 (127.4, 332.3) | 10.7 (-0.5, 19.9) | 0.5 (-0.7, 1.8) |
| Gujarat | 20.0 (10.2, 32.2) | 207.2 (105.8, 332.0) | 13.7 (2.3, 27.1) | 0.5 (-0.4, 1.5) |
| Haryana | 49.8 (41.4, 60.2) | 474.0 (395.0, 573.1) | 25.4 (16.5, 35.0) | 1.2 (0.4, 1.9) |
| Himachal Pradesh | 53.6 (45.3, 65.6) | 586.4 (496.3, 718.2) | 30.5 (18.5, 42.2) | 1.7 (0.6, 2.7) |
| Jammu & Kashmir | 20.1 (10.7, 34.0) | 224.7 (121.1, 380.0) | 13.9 (1.7, 27.8) | 0.7 (-0.3, 2.0) |
| Jharkhand | 48.4 (40.1, 58.0) | 457.5 (379.4, 548.0) | 24.5 (15.5, 32.7) | 1.4 (0.7, 2.6) |
| Karnataka | 23.4 (13.0, 32.0) | 232.6 (131.3, 319.5) | 14.8 (2.7, 24.2) | 0.6 (-0.2, 1.3) |
| Kerala | 23.1 (12.9, 33.8) | 222.6 (125.2, 325.9) | 11.6 (0.4, 23.4) | 0.6 (-0.3, 1.5) |
| Ladakh | 21.6 (10.8, 31.9) | 250.1 (126.9, 368.8) | 11.6 (1.2, 22.7) | 0.8 (-0.4, 2.2) |
| Lakshadweep | 21.1 (9.9, 32.9) | 223.5 (106.3, 347.3) | 9.9 (-2.1, 24.7) | 0.5 (-0.5, 1.7) |
| Madhya Pradesh | 55.1 (44.2, 64.2) | 566.0 (454.1, 659.5) | 34.7 (24.9, 45.0) | 1.7 (1.1, 2.6) |
| Maharashtra | 22.3 (13.6, 31.9) | 232.7 (141.9, 332.7) | 15.2 (6.6, 25.5) | 0.6 (-0.3, 1.6) |
| Manipur | 21.4 (9.7, 30.6) | 237.6 (108.0, 338.7) | 11.9 (-1.0, 24.2) | 0.7 (-0.5, 1.8) |
| Meghalaya | 21.2 (12.0, 31.2) | 220.8 (125.5, 323.7) | 11.5 (1.7, 23.4) | 0.7 (-0.6, 1.5) |
| Mizoram | 20.4 (10.1, 32.2) | 233.9 (117.0, 368.4) | 13.4 (-0.3, 28.2) | 0.7 (-0.7, 1.8) |
| Nagaland | 24.0 (13.6, 36.5) | 255.3 (144.6, 387.2) | 13.9 (2.6, 26.7) | 0.8 (-0.2, 1.9) |
| Odisha | 57.6 (48.6, 64.5) | 588.6 (497.6, 659.5) | 32.7 (23.1, 45.5) | 1.8 (0.8, 2.7) |
| Puducherry | 24.1 (11.6, 35.5) | 250.2 (121.3, 367.5) | 11.5 (-3.2, 21.4) | 0.6 (-0.5, 1.9) |
| Punjab | 44.1 (35.2, 53.2) | 404.5 (323.6, 487.4) | 20.8 (11.5, 29.1) | 0.9 (0.0, 1.7) |
| Rajasthan | 49.3 (37.7, 57.4) | 535.4 (410.7, 623.6) | 31.9 (19.0, 42.4) | 1.6 (0.6, 2.4) |
| Sikkim | 20.2 (9.2, 28.3) | 226.2 (104.4, 316.7) | 11.0 (-1.2, 22.4) | 0.4 (-0.5, 1.8) |
| Tamil Nadu | 51.2 (42.1, 58.8) | 488.7 (401.6, 560.5) | 28.8 (18.9, 38.0) | 1.3 (0.5, 2.2) |
| Telangana | 24.3 (13.3, 38.6) | 237.0 (130.6, 375.3) | 14.8 (5.5, 27.5) | 0.5 (-0.3, 1.6) |
| Tripura | 39.5 (29.5, 48.2) | 431.4 (322.1, 526.7) | 20.1 (8.9, 31.7) | 1.3 (0.3, 2.1) |
| Uttar Pradesh | 43.8 (36.4, 52.2) | 466.9 (388.2, 555.4) | 29.4 (19.0, 39.4) | 1.5 (0.9, 2.4) |
| Uttarakhand | 33.2 (21.3, 46.8) | 372.0 (239.8, 524.3) | 20.0 (5.7, 33.3) | 0.9 (0.0, 1.9) |
| West Bengal | 19.2 (8.0, 28.4) | 201.1 (84.3, 297.1) | 12.8 (0.7, 22.8) | 0.7 (-0.4, 1.9) |

**Table S2. Reduced prevalence, incidence, antibiotic misuse, and deaths from current vaccination coverage by wealth quintile.**

| Wealth Quintile | Reduced Prevalence (% Reduction) | Reduced Cases per 1000 Children per Year | Reduced Antibiotic Courses per 1000 Children per Year | Reduced Deaths per 1000 Children per Year |
| --- | --- | --- | --- | --- |
| 1 | 32.8 (30.2, 35.2) | 339.7 (312.4, 364.7) | 16.8 (14.2, 19.8) | 1.4 (1.0, 1.9) |
| 2 | 33.5 (31.1, 35.9) | 350.8 (325.6, 375.6) | 20.8 (17.0, 24.0) | 1.3 (0.9, 1.8) |
| 3 | 33.5 (30.7, 36.3) | 346.3 (317.3, 374.6) | 22.3 (19.0, 25.2) | 1.0 (0.7, 1.4) |
| 4 | 33.8 (30.6, 36.4) | 340.4 (309.2, 366.3) | 24.3 (20.7, 28.2) | 0.7 (0.5, 1.1) |
| 5 | 34.3 (31.6, 36.6) | 356.5 (328.7, 381.1) | 10.9 (7.1, 13.9) | 0.4 (0.2, 0.7) |
