# Supplementary figures and images for: Effect of rotavirus vaccination on the burden of rotavirus disease and associated antibiotic use in India: A dynamic agent-based simulation analysis
Source: Vaccine. 2024 Sep 17;42(22):None. doi: 10.1016/j.vaccine.2024.126211 (PMC11385704; doi:10.1016/j.vaccine.2024.126211)

Rotavirus ABM Graphs Depicting Median Results from Simulations of the years 2010-2022


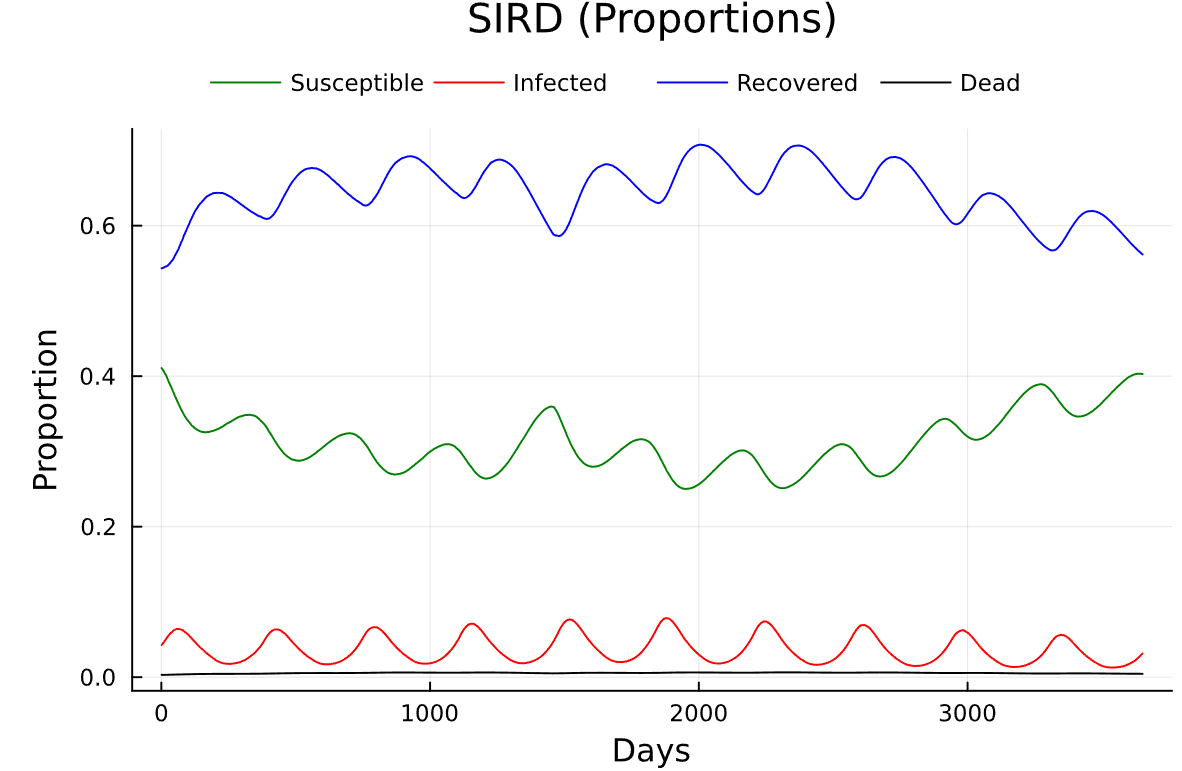


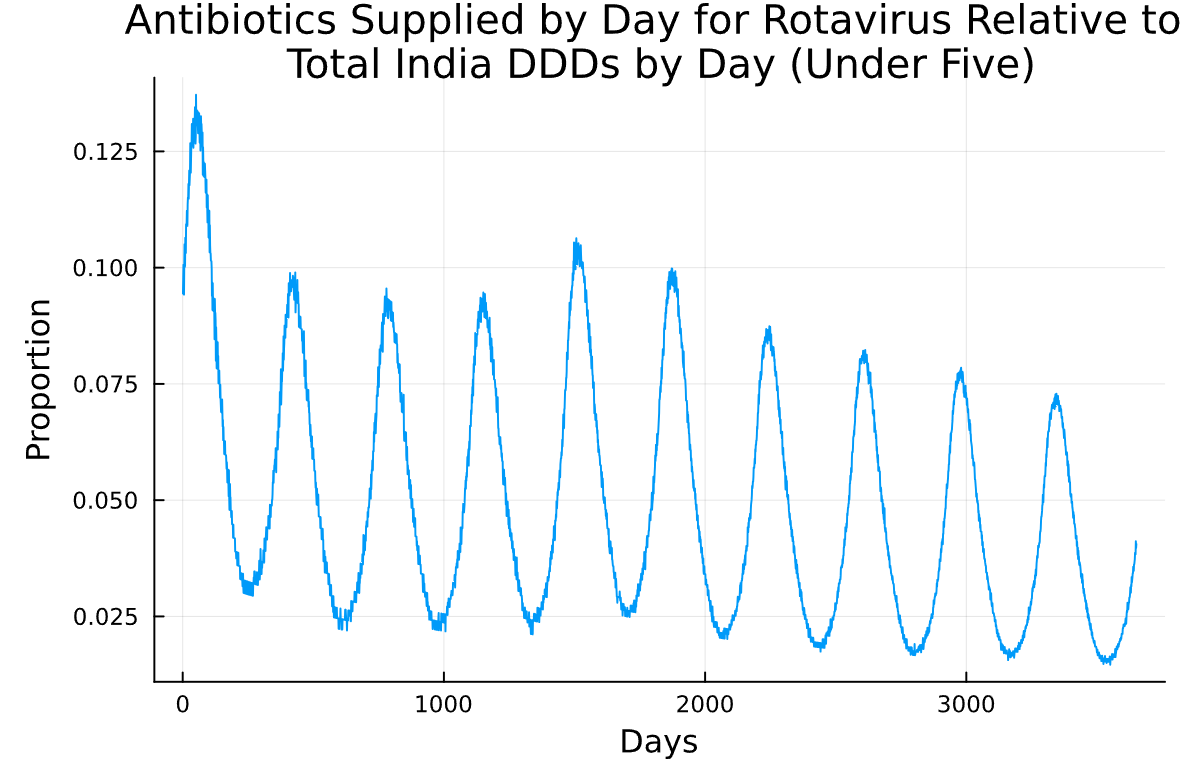


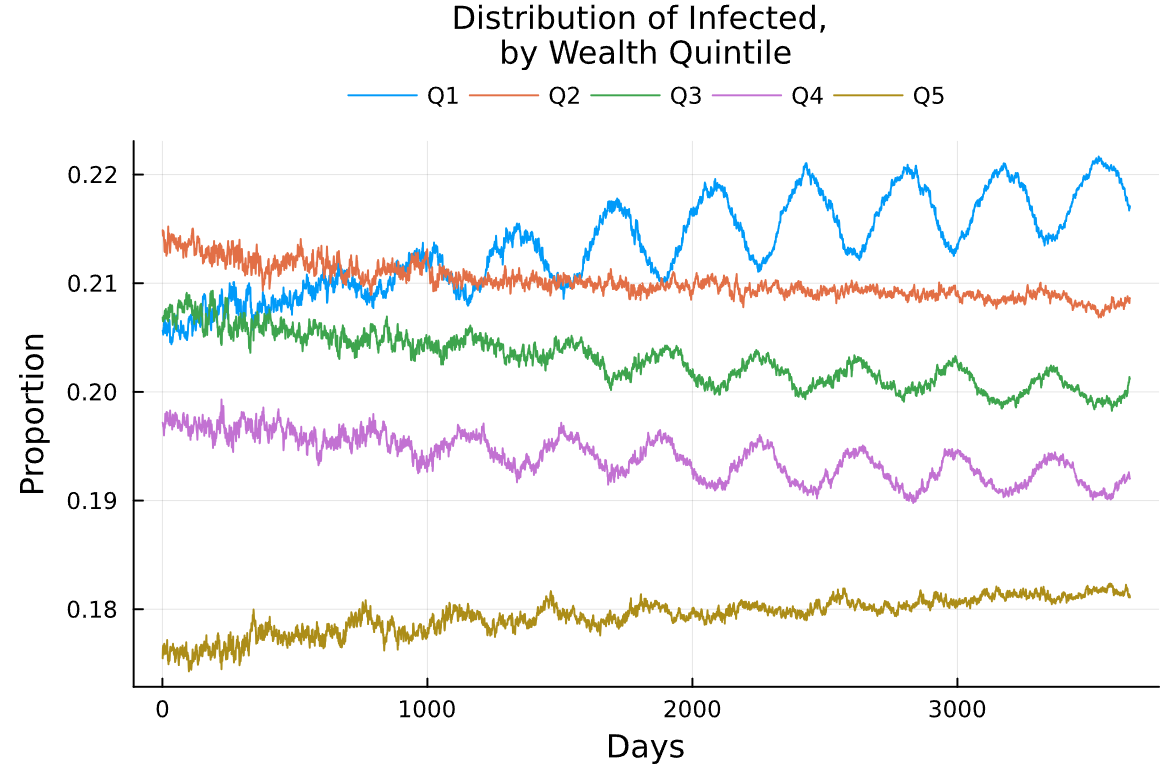

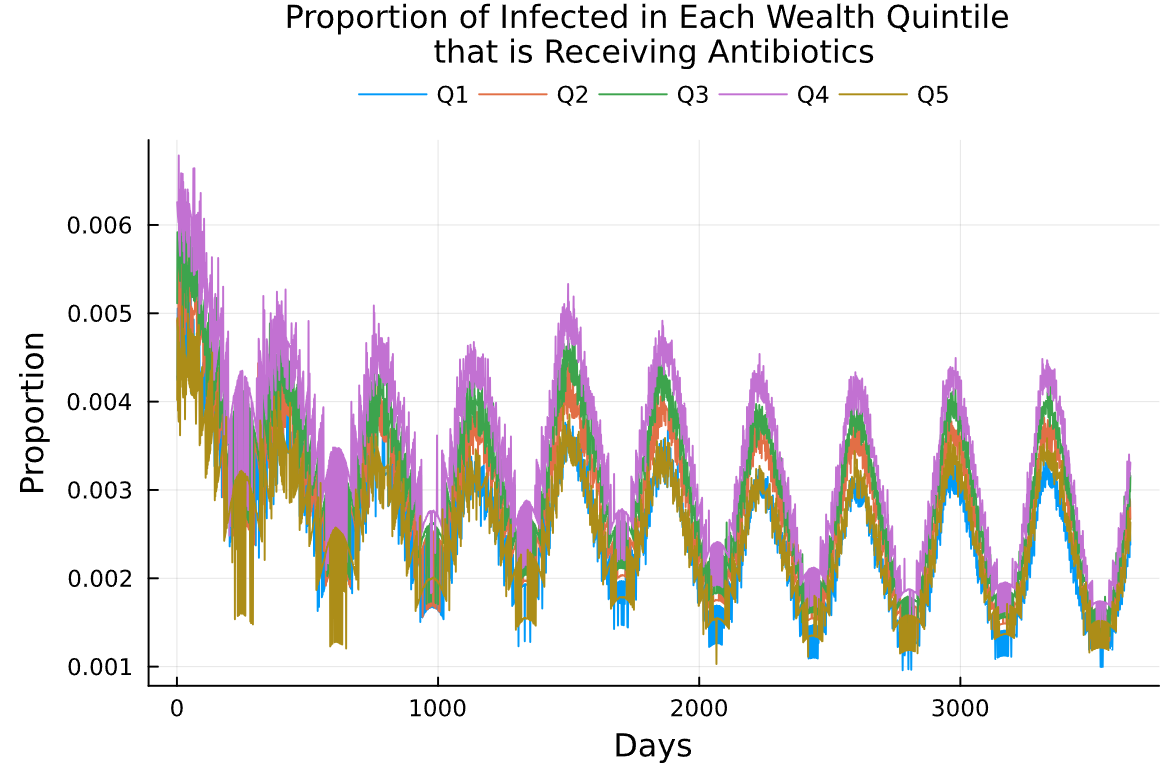


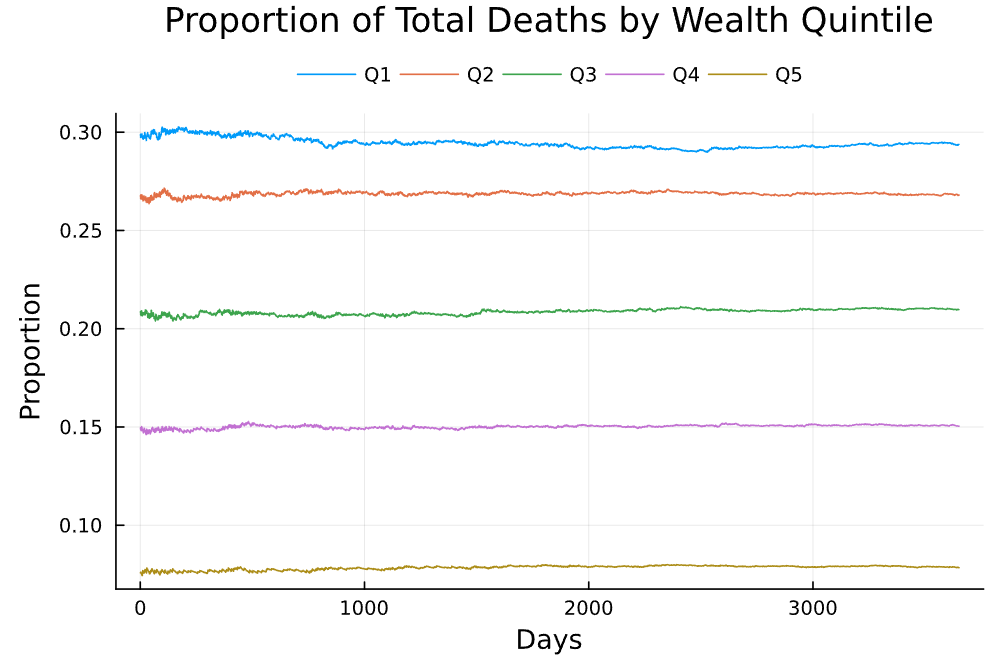

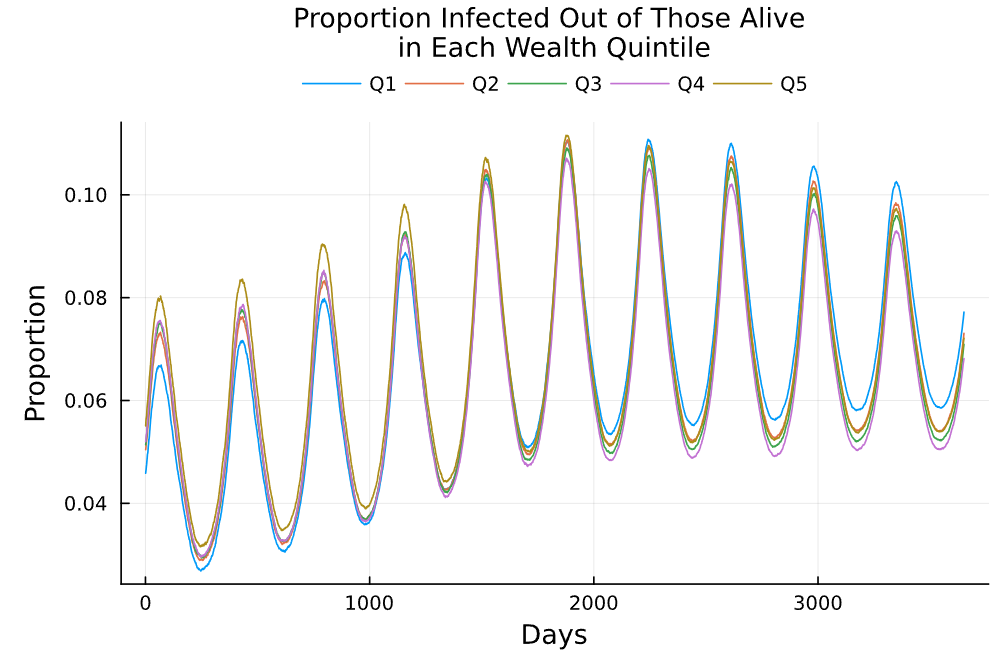

Supplement: Supplementary material 2 — Rotavirus ABM graphs depicting median results from simulation of the years 2010-2022. [file mmc2.docx]
